# Attenuation of microglial aryl hydrocarbon receptor alters astrocyte activation and chronic pain sensitization in aging

**DOI:** 10.1515/biol-2025-1261

**Published:** 2026-02-02

**Authors:** Jia-Xiao Sun, Ying Huang, Juan-Juan Zheng, Wen-Qin Xie

**Affiliations:** Department of Anesthesiology, Quanzhou First Hospital, Fujian Medical University, Quanzhou, 362000, China; Department of Medical Record, Quanzhou First Hospital, Fujian Medical University, Quanzhou, 362000, China

**Keywords:** aryl hydrocarbon receptor, astrocytes, chronic neuropathic pain, microglia, spinal dorsal horn

## Abstract

This study aims to examine differences in aryl hydrocarbon receptor (AHR) expression between microglia in aged and adult mice and to investigate the impact of microglial AHR attenuation on chronic pain sensitization. Immunofluorescence staining was performed to assess AHR expression in microglia. BV2 microglial cells were treated with lipopolysaccharides (LPS), the AHR agonist FICZ, and the AHR antagonist CH223191. The resulting supernatant was used to culture C8-DIA astrocytes, and inflammatory factor levels were quantified using quantitative real-time polymerase chain reaction. AHR expression in spinal dorsal horn microglia was significantly lower in aged mice compared to adult mice. Furthermore, microglial AHR expression was found to regulate astrocyte activation. AHR expression in spinal dorsal horn microglia is markedly reduced in aged mice. Activated microglia with diminished AHR expression induce astrocytes more strongly and enhance astrocyte-mediated inflammation, contributing to prolonged hyperalgesia in aged mice.

## 1 Introduction

Chronic pain is defined as pain that persists for more than three months – exceeding the expected tissue healing period – and is associated with actual or potential tissue damage [1], [2], [3]. It is a major public health issue, and evidence indicates that its prevalence increases with age [1], [2], [3]. Epidemiological studies report that chronic pain affects 52.8 % of older adults in the United States, 57.5 % in Germany, and 60.2 % in China, despite relatively low rates of healthcare utilization [3].

Neuropathic pain (NPP), a common subtype of chronic pain, results from injury or disease affecting the somatosensory nervous system [4]. According to World Health Organization estimates, NPP affects up to 10 % of the general population [5]. Research by Tozza et al. indicates that its incidence increases markedly with aging [6]. However, the underlying pathogenic mechanisms remain insufficiently understood, posing major challenges for preventive and treatment.

With global population aging accelerating, chronic pain – particularly NPP – has gained increasing clinical and research attention. Current evidence suggests that both peripheral and central sensitization contribute to NPP development and are associated with inflammatory mediators released after nerve injury [7], ectopic neuronal discharge [8], and activation of spinal glial cells [9]. Among these mechanisms, neuroinflammation driven by glial cell activation is considered a central factor in the onset and maintenance of central sensitization and neuropathic pain [10].

Conclusions drawn from animal models indicate that in mice with chronic constriction injury (CCI) of the sciatic nerve, both microglia and astrocytes in the spinal dorsal horn become activated, with microglia activation occurring predominantly in the early phase and astrocytic activation in the later phase [11]. This pattern of activation has prompted investigations into the regulatory interactions between microglia and astrocytes. Specifically, upon nerve injury, microglia are rapidly activated and function as primary initiators of neuropathic pain by releasing pro-inflammatory cytokines such as interleukin-1β (IL-1β) and tumor necrosis factor-α (TNF-α) [12]. Subsequently, astrocytes undergo persistent activation and play a more crucial role in the maintenance of chronic pain by amplifying neuroinflammation through factors like C–C motif chemokine ligand 2 (CCL2) and nitric oxide synthase 2 (NOS2) [13]. The communication from microglia to astrocytes is thus considered a pivotal event in the transition from acute to chronic pain states. Among the various regulatory targets, the aryl hydrocarbon receptor (AHR) has been identified as being closely associated with aging [14]. AHR is a transcription factor activated by ligands, expressed in the immune system, and plays a key role in regulating immune function and inflammatory responses [15], 16]. The expression of AHR in microglia is involved in maintaining the balance between anti-inflammatory and pro-inflammatory functions [17]. To pharmacologically probe this microglia-astrocyte axis, we employed several key compounds. Lipopolysaccharide (LPS), a potent toll-like receptor 4 agonist, was used to robustly activate microglia and model neuroinflammation *in vitro*. 6-Formylindolo [3,2-*b*] carbazole (FICZ), a high-affinity endogenous ligand of AHR, was utilized to specifically activate the AHR pathway. Conversely, CH223191, a selective AHR antagonist, was applied to inhibit AHR signaling.

Furthermore, studies have demonstrated that in mouse models of multiple sclerosis-experimental autoimmune encephalomyelitis, knockout of microglial AHR leads to an upregulation of astrocyte-derived inflammatory factors, resulting in exacerbated neuroinflammation [18]. Given these findings, the present study aims to elucidate the effects of microglial activation on astrocytes and its role in the pathophysiology of chronic neuropathic pain.

## 2 Materials and methods

### 2.1 Experimental animals

This study utilized specific pathogen-free (SPF) male C57BL/6 mice, aged 2 months (weighing 25–30 g) and 18 months (weighing 30–35 g), purchased from Shanghai SIPPE-BK Lab Animal Co., Ltd., China. The mice were housed under controlled conditions, with a temperature of 23–25 °C, relative humidity of 45–55 %, and a 12-h light-dark cycle. All animals possessed valid quarantine certificates, and the study protocol was approved by the Laboratory Animal Management Institution of Shanghai Jiao Tong University School of Medicine.

**Ethical approval:** The research related to animal use has been complied with all the relevant national regulations and institutional policies for the care and use of animals, and has been approved by the Ethics Committee of Quanzhou First Hospital (Approval Number: 20230712423, Date: February 2nd 2023).

### 2.2 Main reagents

The primary reagents used in this study included a rabbit recombinant anti-Iba1 antibody (Abcam, UK), an AHR complex antibody (Invitrogen, USA), lipopolysaccharides (LPS) (Sigma-Aldrich, USA), as well as the AHR agonist FICZ and AHR antagonist CH223191 (Selleck, USA).

### 2.3 Construction of sciatic nerve constriction pain model

Mice were anesthetized via intraperitoneal injection of 0.5 % pentobarbital sodium. The left hind limb was immobilized, and an incision was made in the skin posterior to the left femur. Muscles were carefully dissected to expose and isolate the sciatic nerve. The sciatic nerve was ligated at four sites using absorbable surgical sutures, with 1–2 mm intervals between adjacent ligatures. The degree of ligation was adjusted based on the observation of mild twitching in the ipsilateral leg muscles. The skin was sutured after ligation. Mice in the sham group underwent the same surgical procedure without sciatic nerve ligation. A total of 16 mice underwent CCI surgery. All animals were monitored daily for general health and any signs of pain-related behavior, including autotomy. While mild self-directed behaviors (e.g., brief licking or biting of the ipsilateral hind paw) were occasionally observed in some CCI-operated mice, no severe self-mutilation requiring early euthanasia occurred in this cohort.

### 2.4 Immunofluorescence staining

Mice were anesthetized with 0.5 % pentobarbital sodium via intraperitoneal injection and euthanized. They were then perfused with normal saline, followed by 4 % paraformaldehyde fixation. After perfusion, spinal cord segments (L4–L6) were collected and post-fixed in 4 % paraformaldehyde at 4 °C overnight. The tissues were sequentially cryoprotected in graded sucrose solutions (10 %, 20 %, and 30 %) until sinking at each step. After cryoprotection, samples were embedded in optimal cutting temperature (OCT) compound and sectioned into 20-μm slices.

Sections were permeabilized with 1 % PBST for 30 min and blocked with an immunofluorescence blocking buffer for 1 h. Primary antibodies (IBA1 1:1,000; AHR 1:200) were applied and incubated overnight at 4 °C, followed by incubation with secondary antibodies (1:1,000) for 2 h at room temperature. After each incubation, sections were washed three times with 0.3 % PBST for 10 min. Finally, the slides were mounted, counterstained with DAPI, and examined under a fluorescence or confocal microscope.

### 2.5 Cell culture and treatment

Microglia (BV2) and astrocytic (C8-DIA) cell lines were cultured in specialized culture media under standard conditions. Drug treatments were administered when cells reached approximately 80 % confluence in 10 cm culture dishes or 6-well plates. Cells were treated with LPS at concentrations of 0 μg/ml, 0.01 μg/ml, 0.1 μg/ml, and 1 μg/ml; FICZ at concentrations of 0 nM, 10 nM, 100 nM, and 1 µM; or CH223191 at concentrations of 0 nM, 10 nM, 1 µM, 10 µM, and 100 µM. The specific cleaning process is as follows: after BV2 microglial cells were treated with LPS, FICZ or CH223191 for the predetermined time, the original culture medium containing the drugs was discarded. Subsequently, the cells were washed three times thoroughly with pre-warmed sterile PBS (phosphate buffered saline). Each time, an adequate amount of PBS was added, the culture plate was gently shaken and the supernatant was completely aspirated to ensure the maximum removal of unbound drug molecules. After the third PBS wash, we replaced it with brand-new DMEM basal medium without any drugs or serum and continued to culture for 24 h to collect the conditioned medium. Following treatment, the supernatant was filtered through a 0.22 µm filter for subsequent C8-DIA culture, and cellular RNA was extracted using commercial kits for qRT-PCR analysis.

### 2.6 Quantitative real-time polymerase chain reaction (qRT-PCR)

Total RNA was extracted from cells, and RNA concentration was measured using a spectrophotometer. Complementary DNA (cDNA) was synthesized via reverse transcription and subsequently amplified using a real-time fluorescence quantitative PCR instrument. Glyceraldehyde-3-phosphate dehydrogenase (GAPDH) served as an internal reference to normalize the cycle threshold (Ct) values of target genes. Relative gene expression levels were analyzed using the 2ˆ(−ΔΔCt) method. The primer sequences used in the analysis are shown in Table 1.

**Table 1:** qRT-PCR primer sequences.

Gene		Primer sequence
AHR	Forward	AGC​CGG​TGC​AGA​AAA​CAG​TAA
	Reverse	AGG​CGG​TCT​AAC​TCT​GTG​TTC
CYP1A1	Forward	GAC​CCT​TAC​AAG​TAT​TTG​GTC​GT
	Reverse	GGT​ATC​CAG​AGC​CAG​TAA​CCT
CYP1B1	Forward	CAC​CAG​CCT​TAG​TGC​AGA​CAG
	Reverse	GAG​GAC​CAC​GGT​TTC​CGT​TG
GAPDH	Forward	AGG​TCG​GTG​TGA​ACG​GAT​TTG
	Reverse	TGT​AGA​CCA​TGT​AGT​TGA​GGT​CA
NOS2	Forward	GTT​CTC​AGC​CCA​ACA​ATA​CAA​GA
	Reverse	GTG​GAC​GGG​TCG​ATG​TCA​C
IL-1β	Forward	GCA​ACT​GTT​CCT​GAA​CTC​AAC​T
	Reverse	ATC​TTT​TGG​GGT​CCG​TCA​ACT
TNFα	Forward	CCC​TCA​CAC​TCA​GAT​CAT​CTT​CT
	Reverse	GCT​ACG​ACG​TGG​GCT​ACA​G
IL-6	Forward	TAG​TCC​TTC​CTA​CCC​CAA​TTT​CC
	Reverse	TTG​GTC​CTT​AGC​CAC​TCC​TTC
CCL2	Forward	TTA​AAA​ACC​TGG​ATC​GGA​ACC​AA
	Reverse	GCA​TTA​GCT​TCA​GAT​TTA​CGG​GT

### 2.7 Statistical methods

Data analysis and graphing were performed using GraphPad Prism 9.0 software. Mechanical pain measurement data were analyzed using a two-way analysis of variance (ANOVA) with repeated measures, followed by a least significant difference (LSD) test for pairwise comparisons. The selection of the LSD test for post hoc analysis was based on our focus on pre-planned, specific pairwise comparisons that are essential to the hypothesis of this study, providing adequate power for detecting biologically relevant effects. Cellular quantitative real-time PCR results were analyzed using a one-way analysis of variance, while immunofluorescence data were analyzed using an independent sample *t*-test. Values are expressed as mean ± standard error of mean (mean ± SEM). Differences were considered statistically significant at *p* < 0.05.

## 3 Results

### 3.1 Persistent hyperalgesia in aged mice with chronic neuropathic pain

**Figure 1:**
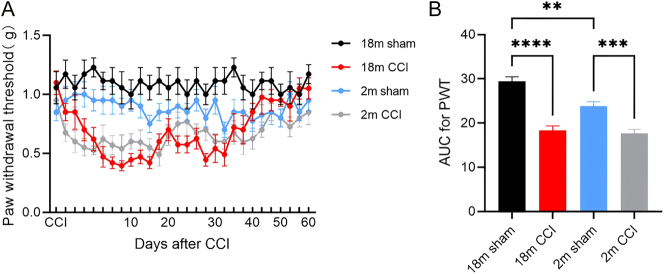
Mechanical pain threshold in 2-month-old and 18-month-old mice (*n* = 8) (A) time – dependent changes in the mechanical pain threshold measured continuously over 60 days using von Frey filaments. (B) Area under the curve (AUC) analysis of baseline mechanical pain threshold changes in 2-month-old and 18-month-old mice. The groups include the 18 m sham: 18-month-old sham group, 18 m CCI: 18-month-old sciatic nerve constriction pain group, 2 m sham: 2-month-old sham group, 2 m CCI: 2-month-old sciatic nerve constriction pain group. Statistical significance is indicated as follows: *p < 0.05, **p < 0.01, ***p < 0.001, ****p < 0.0001. Data are expressed as mean ± SEM.

CCI models were generated in both 2-month-old and 18-month-old mice, and mechanical pain thresholds were evaluated using von Frey filaments. Following CCI, both age groups exhibited a significant decline in mechanical threshold compared with sham controls, confirming successful model induction (Figure 1A and B) (2-month-old sham vs. CCI, *p* = 0.0005; 18-month-old sham vs. CCI, *p* < 0.0001). Notably, baseline thresholds were significantly higher in 18-month-old mice compared with 2-month-old mice (Figure 1A and B) (*p* = 0.0024). After CCI, mechanical sensitivity in 2-month-old mice returned to baseline by approximately day 20, whereas 18-month-old mice required up to 40 days for recovery. Together, these findings indicate that aged mice develop more persistent hyperalgesia and slower recovery following neuropathic injury compared with young mice.

### 3.2 Reduced AHR expression in spinal dorsal horn microglia of aged mice

**Figure 2:**
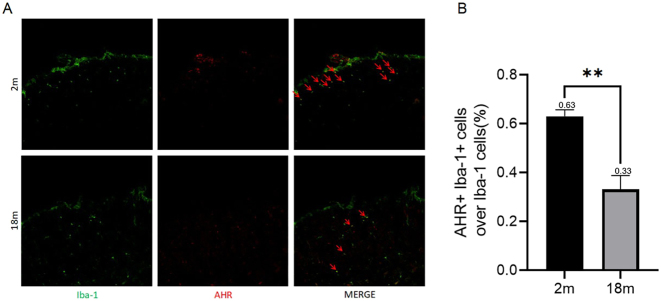
Immunofluorescence analysis of microglia and AHR expression in the spinal dorsal Horn of 2-month-old and 18-month-old mice (A) immunofluorescence detection of microglia and AHR co-labeling in spinal dorsal horn of adult and aged mice. Green fluorescence represents microglial marker Iba1, while red fluorescence represents AHR expression. (B) Percentage of AHR-expressing microglia relative to total microglia in adult and aged mouse spinal dorsal horn. Groups include the 2 m: 2-month-old blank group, 18 m: 18-month-old blank group. Statistical significance is indicated as follows: *p < 0.05, **p < 0.01. Data are expressed as mean ± SEM.

To investigate potential differences in AHR expression in spinal dorsal horn microglia between aged and adult mice, spinal cord segments (L4–L6) were collected following euthanasia under anesthesia. Immunofluorescence staining for IBA1 and AHR revealed a significant reduction in AHR expression in spinal dorsal horn microglia of 18-month-old mice compared to 2-month-old mice (Figure 2) (*p* = 0.0087). These results indicate that diminished AHR expression in aged mice may contribute to heightened microglial and astrocytic activation, potentially leading to prolonged hyperalgesia in the context of chronic neuropathic pain.

### 3.3 Enhanced astrocyte activation and inflammation mediated by microglia with low AHR expression

To further investigate the influence of microglial activation on astrocyte activation and the regulatory role of the microglial AHR pathway in astrocyte-mediated inflammation, *in vitro* experiments were conducted using the BV2 microglial and C8-DIA astrocyte cell lines.

#### 3.3.1 Effects of different LPS concentrations on microglial activation

**Figure 3:**
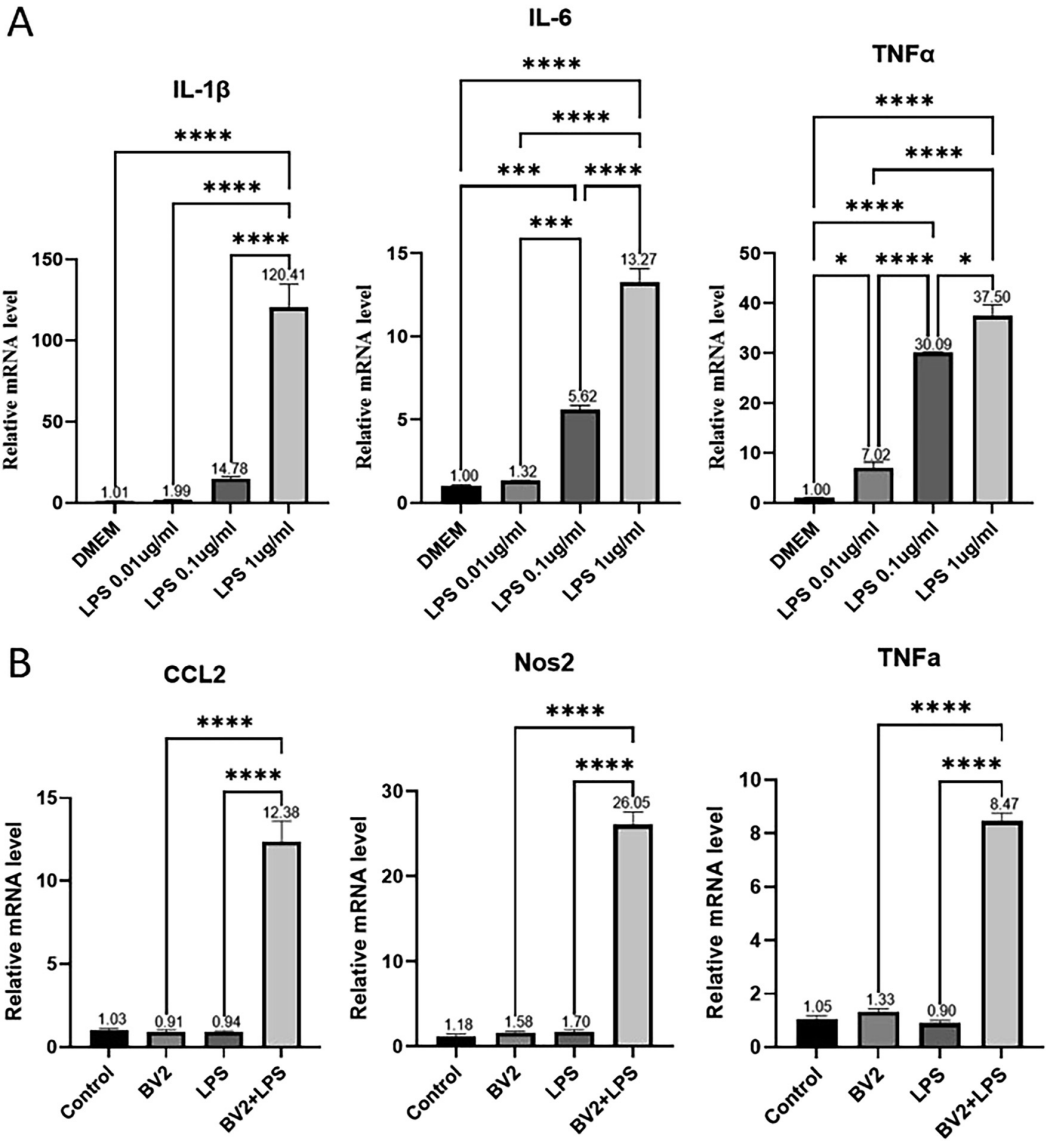
Relative mRNA levels of glial cell inflammatory factors after LPS treatment (A) transcription levels of microglial activation-related genes after 24 h treatment with different concentrations of LPS (0 μg/ml, 0.01 μg/ml, 0.1 μg/ml, 1 μg/ml). (B) Transcription levels of astrocyte inflammatory factors after 24 h treatment with control medium, microglial supernatant (BV2), LPS, or activated microglial supernatant (BV2 + LPS). Statistical significance is indicated as follows: *p < 0.05, **p < 0.01, ***p < 0.001, ****p < 0.0001. Data are expressed as mean ± SEM.

Microglial cells were stimulated with LPS at different concentrations (0, 0.01, 0.1, and 1 μg/ml) for 24 h to induce activation, followed by RNA extraction for qRT-PCR analysis. Stimulation with 1 μg/ml LPS resulted in the highest expression levels of pro-inflammatory cytokine mRNA, including interleukin-1β (IL-1β), interleukin-6 (IL-6), and tumor necrosis factor α (TNF-α), with all showing significant upregulation (*p* < 0.0001) (Figure 3A). Based on these findings, 1 μg/ml LPS was selected as the optimal working concentration for microglial activation in subsequent experiments.

#### 3.3.2 Activation of microglia exacerbates astrocyte-mediated inflammatory responses

To assess the influence of activated microglia on astrocyte-mediated inflammation, supernatants collected from microglia stimulated with 1 μg/ml LPS for 24 h were filtered through a 0.22 µm membrane and applied to astrocyte culture. After 24 h of exposure, astrocyte RNA was extracted for qRT-PCR analysis. Compared with astrocytes treated with non-activated microglial supernatant (BV2), those exposed to activated microglial supernatant (BV2 + LPS) exhibited significantly increased expression of inflammatory mediators, including CCL2, NOS2, and TNF-α (*p* < 0.0001 for all) (Figure 3B). These findings suggest that activated microglia enhance astrocyte inflammatory responses.

#### 3.3.3 Effects of AHR antagonists on microglial AHR expression

**Figure 4:**
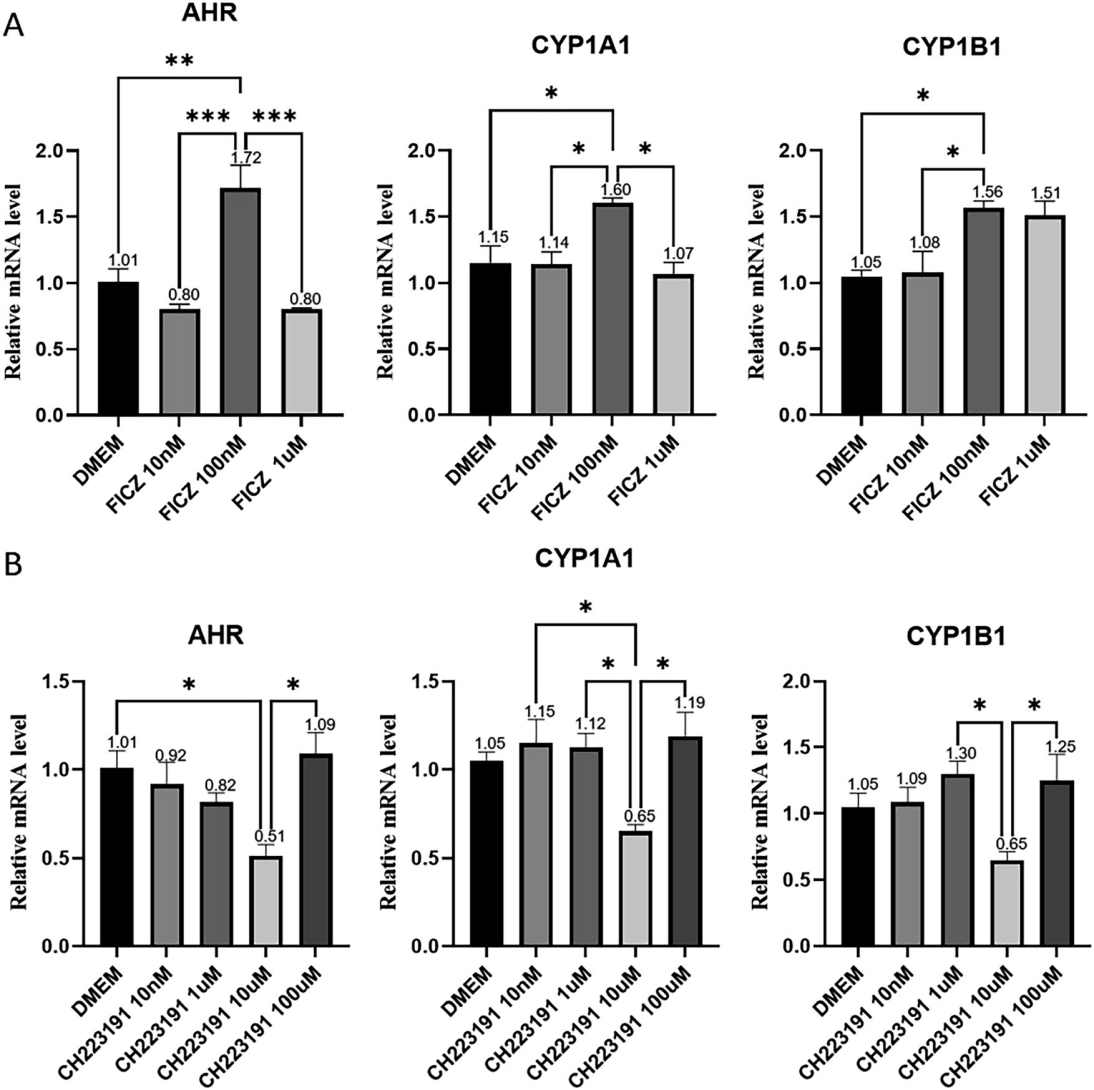
Relative mRNA levels of microglial AHR pathway after FICZ/CH223191 treatment (A) transcription levels of AHR and its downstream targets, CYP1A1 and CYP1B1, after 24-h treatment with different concentrations of the AHR agonist FICZ (0 nM, 10 nM, 100 nM, 1 µM). (B) Transcription levels of AHR and its downstream targets, CYP1A1, and CYP1B1, after 12-h treatment with different concentrations of the AHR antagonist CH223191 (0 nM, 10 nM, 1 uM, 10 µM, 100 µM). Statistical significance is indicated as follows: *p < 0.05, **p < 0.01, ***p < 0.001. Data are expressed as mean ± SEM.

To investigate the regulation of the microglial AHR pathway, cells were treated with different concentrations of the AHR agonist FICZ (0 nM, 10 nM, 100 nM, 1 µM) for 24 h, followed by RNA extraction for qRT-PCR analysis. Treatment with 100 nM FICZ produced the most significant upregulation of AHR (*p* = 0.0046) and increased expression of downstream target cytochrome P450 family 1 subfamily A member 1 (CYP1A1) (*p* = 0.0352) and cytochrome P450 family 1 subfamily B member 1 (CYP1B1) (*p* = 0.0299) (Figure 4A). Accordingly, 100 nM FICZ was selected for subsequent microglial AHR activation experiments.

To inhibit the AHR pathway, microglia were treated with varying concentrations of the AHR antagonist CH223191 (0 nM, 10 nM, 1 µM, 10 µM, 100 µM) for 12 h qRT-PCR analysis showed that 10 µM CH223191 induced the most significant downregulation of AHR (*p* = 0.0287) and reduced expression of CYP1A1 and CYP1B1 (Figure 4B). Therefore, 10 µM CH223191 was used for subsequent AHR inhibition studies.

#### 3.3.4 Effects of AHR pathway activation and inhibition on astrocyte inflammatory levels following microglial activation

**Figure 5:**
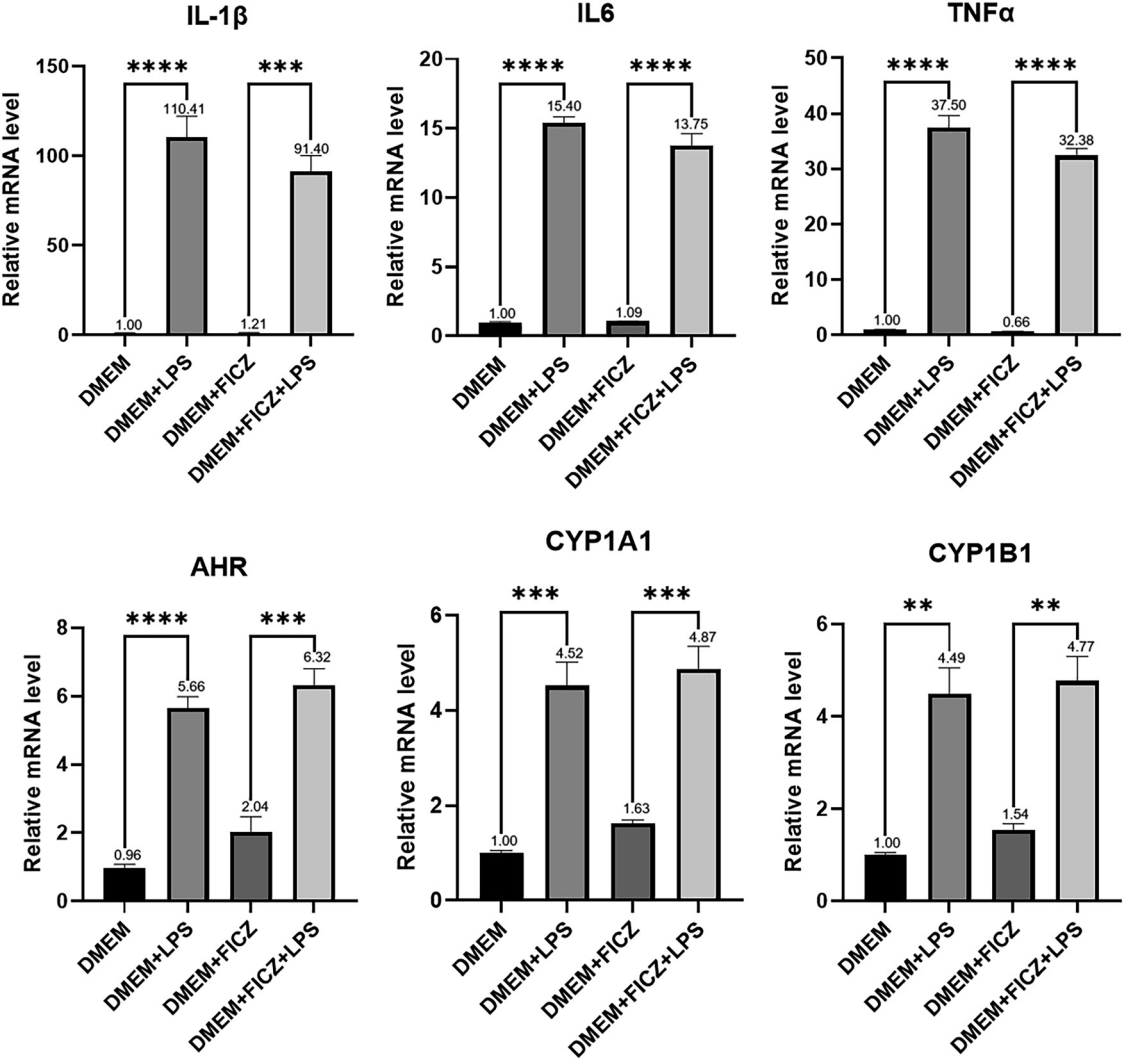
Relative mRNA levels of microglial inflammatory factors and AHR pathway after LPS/FICZ treatment transcription levels of inflammatory factors and AHR pathway components after 24-h treatment with control medium (DMEM), LPS (DMEM + LPS), the AHR agonist (DMEM + FICZ), or a combination of LPS and FICZ (DMEM + FICZ + LPS). Statistical significance is indicated as follows: *p < 0.05, **p < 0.01, ***p < 0.001, ****p < 0.0001. Data are expressed as mean ± SEM.

In this experimental phase, microglial cells were treated with 1 µg/ml LPS and 100 nM FICZ to activate both microglia and the AHR pathway. Following treatment, RNA was extracted from the microglia for qRT-PCR analysis. The results demonstrated that compared to control (DMEM), microglial activation (DMEM + LPS) significantly upregulated the expression of inflammatory factors IL-1β (*p* < 0.0001), IL-6 (*p* < 0.0001), TNFα (*p* < 0.0001), and AHR pathway components AHR (*p* < 0.0001), CYP1A1 (*p* = 0.0004), and CYP1B1 (*p* = 0.0011). Compared to activated microglia (DMEM + FICZ), activated microglia with AHR pathway activation (DMEM + LPS + FICZ) showed decreased expression of inflammatory factors (IL-1β (*p* < 0.0001), IL-6 (*p* < 0.0001), and TNFα (*p* < 0.0001)) and increased expression of AHR pathway components AHR (*p* = 0.0002), CYP1A1 (*p* = 0.0008), and CYP1B1 (*p* = 0.0018), in Figure 5. These results indicate that while activated microglia enhance inflammatory responses, AHR pathway activation in these cells can reduce pro-inflammatory factor secretion and mitigate inflammatory responses.

**Figure 6:**
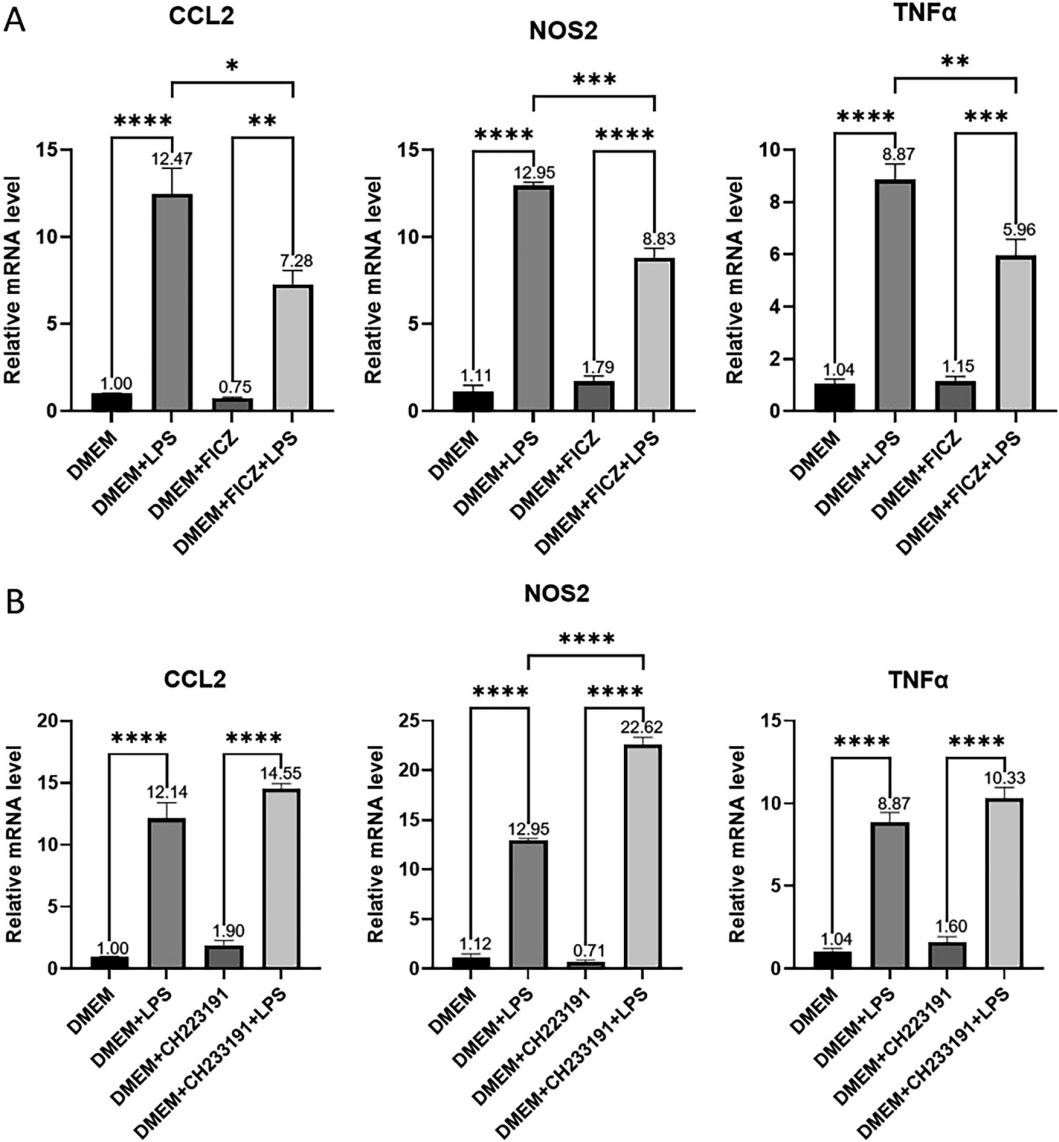
Relative mRNA levels of astrocyte inflammatory factors after exposure to microglial supernatant treated with FICZ/CH223191 (A) transcription levels of astrocyte-related genes after 24-h treatment with LPS/FICZ-treated microglial supernatant. (B) Transcription levels of astrocyte-related genes after 24-h treatment with LPS or CH223191-treated microglial supernatant. Statistical significance is indicated as follows: *p < 0.05, **p < 0.01, ***p < 0.001, ****p < 0.0001. Data are expressed as mean ± SEM.

Subsequently, astrocytes were cultured for 24 h with filtered supernatant (0.22 µm) from the variously treated microglia, followed by RNA extraction from the astrocytes for qRT-PCR. The results indicated that compared to treatment with normal microglial supernatant (DMEM), treatment with activated microglial supernatant (DMEM + LPS) significantly upregulated astrocyte inflammatory factors CCL2 (*p* < 0.0001), NOS2 (*p* < 0.0001), and TNFα (*p* < 0.0001). Compared to treatment with activated microglial supernatant (DMEM + LPS), treatment with supernatant from activated microglia with AHR pathway activation (DMEM + FICZ + LPS) significantly downregulated astrocyte inflammatory factors CCL2 (*p* = 0.0102), NOS2 (*p* = 0.0002), and TNFα (*p* = 0.0076) (Figure 6). These findings indicate that activated microglia can exacerbate astrocyte inflammatory levels, while AHR pathway activation in activated microglia can attenuate the inflammatory response in astrocytes.

#### 3.3.5 Effects of AHR pathway inhibition on astrocyte inflammatory levels following microglial activation

**Figure 7:**
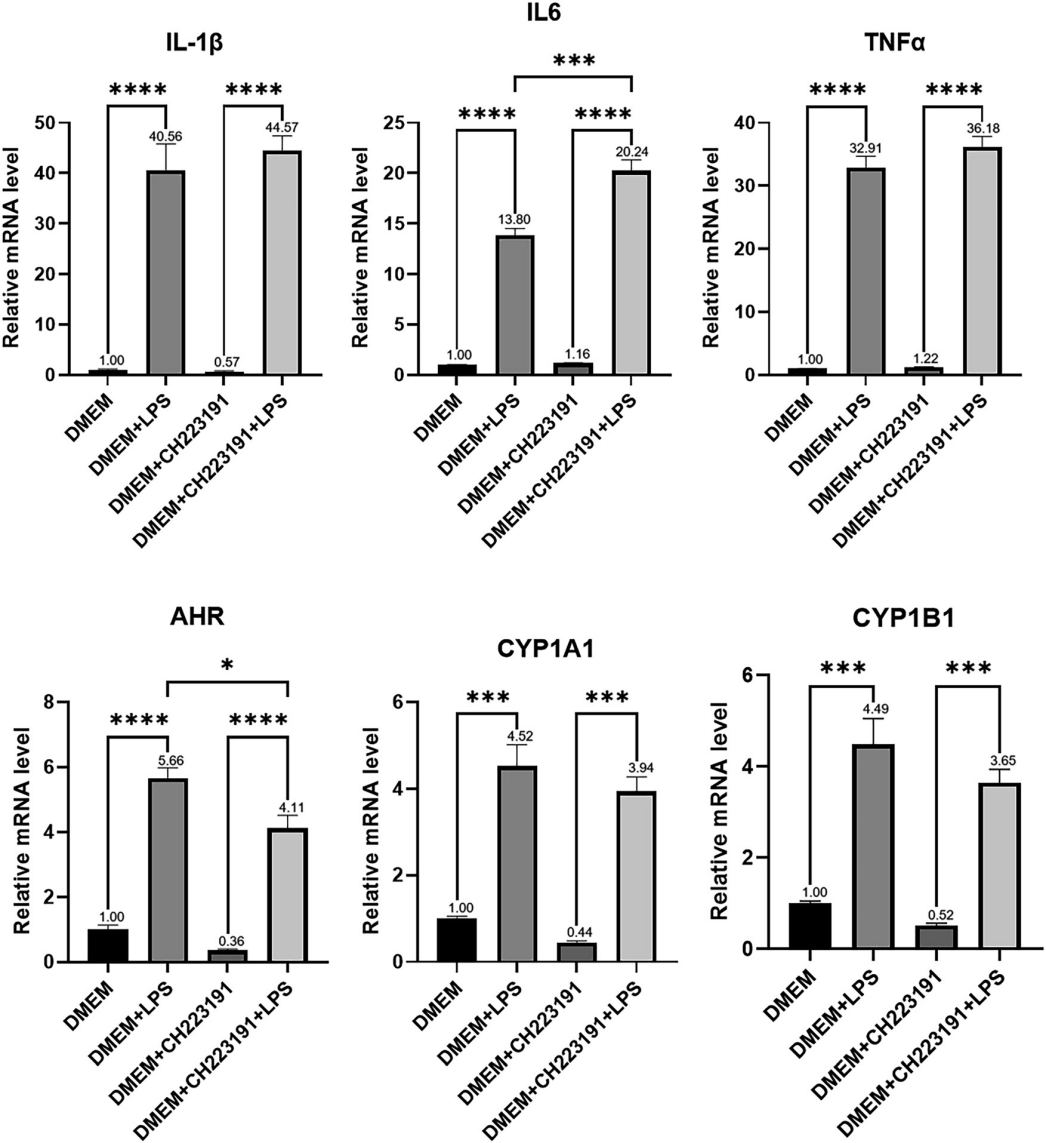
Relative mRNA levels of microglial inflammatory factors and AHR pathway after LPS/CH223191 treatment transcription levels of inflammatory factors and AHR pathway components after 12-h treatment with control medium (DMEM), LPS (DMEM + LPS), the AHR antagonist (DMEM + CH223191), LPS + AHR antagonist (DMEM + LPS + CH223191). Statistical significance is indicated as follows: *p < 0.05, **p < 0.01, ***p < 0.001, ****p < 0.0001. Data are expressed as mean ± SEM.

Microglial cells were treated with 1 μg/ml LPS for 24 h and 10 µM CH223191 for 12 h to activate microglia and inhibit the AHR pathway, respectively, followed by RNA extraction from the microglia for qRT-PCR. The results indicated that compared to the control (DMEM), microglial activation (DMEM + LPS) significantly upregulated the expression of inflammatory factors IL-1β (*p* < 0.0001), IL-6 (*p* < 0.0001), TNFα (*p* < 0.0001), and AHR pathway components AHR (*p* < 0.0001), CYP1A1 (*p* = 0.0002), and CYP1B1 (*p* = 0.0002). In contrast, microglial activation with AHR pathway inhibition (DMEM + LPS + CH223191) led to upregulation of IL-6 expression (*p* = 0.0005), increased expression of IL-1β and TNFα, downregulation of AHR expression (*p* = 0.0151), and decreased expression of CYP1A1 and CYP1B1 (Figure 7). These results indicate that while activated microglia enhance inflammatory responses, inhibition of the AHR pathway in activated microglia promotes the release of pro-inflammatory factors, thereby exacerbating the inflammatory responses.

After culturing astrocytes for 24 h with filtered supernatant (0.22 µm) from the variously treated microglia, RNA was extracted from the astrocytes for qRT-PCR analysis. The results revealed that compared to treatment with normal microglial supernatant (DMEM), treatment with activated microglial supernatant (DMEM + LPS) significantly upregulated astrocyte inflammatory factors CCL2 (*p* < 0.0001), NOS2 (*p* < 0.0001), and TNFα (*p* < 0.0001). Furthermore, compared to treatment with activated microglial supernatant (DMEM + LPS), treatment with supernatant from activated microglia with AHR pathway inhibition (DMEM + CH223191 + LPS) significantly upregulated NOS2 expression (*p* < 0.0001), with increased CCL2 and TNFα expression (Figure 6B). These findings suggest that activated microglia can amplify astrocyte inflammation, while inhibiting the AHR pathway in activated microglia further intensifies these inflammatory responses in astrocytes.

## 4 Discussion

This study investigated the role of microglia in NPP, with a particular focus on microglia as a critical entry point. Through the establishment of the CCI model, von Frey filament pain measurement, immunofluorescence, and cellular quantitative real-time PCR experiments, the study delineated the effects and mechanisms by which microglia contribute to NPP. The results underscore the significant role of microglia in NPP, with activated microglia involved in the maintenance of NPP through the regulation of astrocyte activation and inflammatory responses via the AHR pathway. Reduced AHR expression in the spinal dorsal horn microglia of aged individuals diminishes their capacity to regulate astrocyte activation and inflammation, leading to prolonged astrocyte overactivation and more persistent hyperalgesia in aged mice.

Previous seminal studies, such as Rothhammer et al. [19] in experimental autoimmune encephalomyelitis (EAE) models, have established that microglial AHR regulates astrocyte activation and CNS inflammation via ligands like transforming growth factor alpha (TGF-α) and vascular endothelial growth factor b (VEGF-B). Similarly, Lee et al. [20] reported that AHR mediates bidirectional effects in LPS-activated microglia. While these works elegantly delineated AHR’s role in classic neuroinflammatory contexts (e.g., autoimmune demyelination), our study extends this paradigm to age-related chronic pain, a condition with distinct pathophysiology centered on peripheral and central sensitization. Unlike EAE – which involves massive immune cell infiltration and acute attack – CCI-induced NPP models a more clinically prevalent scenario of nerve injury-induced sensitization, where aging is a paramount risk factor. Our work is the first to directly link microglial AHR dysfunction to the prolonged pain hypersensitivity observed in aging, thereby shifting the focus from acute autoimmune inflammation to chronic pain maintenance in the elderly. This contextual innovation is underscored by our data showing that aged mice exhibit sustained mechanical allodynia for over 40 days post-CCI, whereas young adults recover by day 20 (Figure 1A and B). A critical novel insight from our study is the identification of age-dependent reduction in microglial AHR expression as a mechanism underlying chronic pain persistence. While prior research noted that aging influences AHR in neurodegenerative contexts, our results provide direct evidence that physiological aging per se diminishes AHR levels in spinal microglia, which in turn disrupts the microglia-astrocyte axis. Immunofluorescence analysis confirmed significantly lower AHR co-localization with Iba1+ microglia in the spinal dorsal horn of aged mice (Figure 2), indicating that basal AHR tone is compromised with age. This attenuation predisposes aged microglia to a hyperinflammatory state upon activation, as evidenced by enhanced release of pro-inflammatory factors (e.g., IL-1β, TNF-α) and reduced ability to constrain astrocyte-mediated inflammation. Thus, our work moves beyond simply confirming AHR’s involvement in neuroinflammation – it elucidates how natural aging reprograms microglial AHR signaling, leading to a failure in resolving pain-related neuroinflammation. This offers a new mechanism for age-associated vulnerability to chronic pain. Recent investigations have explored the regulatory relationship between microglia and astrocytes, revealing that activated microglia can induce astrocyte activation through multiple pathways and regulate astrocyte inflammatory levels via various targets. The AHR pathway, in particular, has been shown to be closely linked to age-related changes [14]. Research has demonstrated that in multiple sclerosis-experimental autoimmune encephalomyelitis (EAE) mouse models, the knockout of microglial AHR leads to an upregulation of astrocyte inflammatory factors, thereby exacerbating inflammatory responses [18].

To further investigate the role of AHR in microglial activation-induced astrocyte activation and its involvement in neuropathic pain, microglia were treated with the AHR agonist FICZ and the AHR antagonist CH223191. Microglial supernatants were then used to culture astrocytes, and the expression of astrocyte inflammatory factor was observed. The results indicated that activated microglia exacerbate astrocyte inflammatory levels; however, activation of the AHR pathway in activated microglia reduced the secretion of pro-inflammatory factors, thereby alleviating inflammatory responses. In contrast, inhibition of the AHR pathway in activated microglia led to increased release of pro-inflammatory factors, exacerbating the inflammatory response.

While our study provides a functional characterization of microglia–astrocyte crosstalk in the context of aging, the precise molecular intermediates remain to be fully elucidated. Notably, AHR signaling is known to interact with the nuclear factor kappa B (NF-κB) pathway, suggesting a potential mechanism linking microglial activation to astrocyte-mediated inflammatory responses. Our observation that AHR activation suppresses IL-1β and TNF-α expression (Figure 5) is consistent with the mechanism where ligand-bound AHR complexes with NF-κB subunits (e.g., RelB), thereby limiting their transcriptional activity. Furthermore, the subsequent astrocyte activation we observed (characterized by CCL2 and NOS2 upregulation) mirrors the phenotype induced by microglia-derived TGF-α and VEGF-B in EAE models. It is highly probable that the age-associated decline in AHR we identified leads to a disinhibition of NF-κB, resulting in reduced production of neuroprotective factors (like TGF-α) and increased release of pro-inflammatory cytokines. Although we focused on the functional outcomes of this axis in chronic pain, future studies will specifically quantify these intermediate mediators to map the precise molecular trajectory from the aged microglial nucleus to the astrocytic receptor.

### 4.1 Limitations and Future Perspectives

It is important to note a limitation *in vitro* approach. While the use of conditioned medium from microglia is a well-established and valuable method for investigating paracrine signaling, it provides correlative evidence. This experimental model does not fully recapitulate the direct and dynamic cell-to-cell interactions that occur in a co-culture system or *in vivo*. Therefore, our conclusion that microglial AHR signaling modulates astrocyte activation is based on the strong, pharmacologically-induced, and dose-dependent changes observed in astrocyte inflammatory responses upon manipulation of microglial AHR. Furthermore, *in vivo* studies utilizing microglia-specific AHR knockout mice would be essential to validate the role of this signaling axis in chronic pain sensitization within the complex tissue microenvironment. Identifying the specific AHR-regulated soluble factors released by microglia that mediate this cross-talk represents another critical goal for future research.

While this study provides mechanistic insights into the role of microglial AHR in the spinal dorsal horn, it is important to acknowledge its scope. Our findings are primarily confined to the spinal level, which serves as a critical gateway for pain signal processing and central sensitization. However, neuropathic pain is a complex disorder involving a cascade of events along the neuraxis, from peripheral nerve endings and dorsal root ganglia (DRG) to higher brain centers such as the hippocampus and somatosensory cortex. The enhanced and prolonged activation of spinal astrocytes due to age-related decline in microglial AHR, as identified here, likely represents a pivotal amplification step that perpetuates aberrant signaling throughout this pathway. Future investigations examining the expression and function of AHR in microglia and other immune cells at additional sites, including the DRG and supraspinal regions, will be essential to construct a more comprehensive picture of how this receptor influences chronic pain states. Such studies would not only validate the broader significance of our findings but also potentially identify novel therapeutic targets for interrupting the pain cascade at multiple levels.

Another limitation of this study is the use of immortalized cell lines (BV2 and C8-D1A). Although these models were invaluable for their reproducibility and ease of genetic and pharmacological manipulation, they may not fully recapitulate the complex physiological state of primary glial cells *in vivo*. Therefore, our conclusions regarding AHR-mediated microglial regulation of astrocyte activation are based on robust, dose-dependent pharmacological evidence from a reductionist system. Future studies utilizing primary microglial and astrocytic cultures, or direct co-culture systems, will be essential to validate these findings in a more physiologically relevant context. Furthermore, while our study provides compelling correlative and pharmacological evidence supporting the role of microglial AHR in age-dependent pain persistence, a definitive causal link would be strengthened by future *in vivo* genetic interventions. The most direct approach would involve utilizing microglia-specific conditional AHR knockout mice (e.g., Cx3cr1-CreERT2; AHRf/f) to determine if loss of AHR in microglia is sufficient to recapitulate the prolonged pain phenotype observed in aged mice. Conversely, rescue experiments aimed at overexpressing AHR specifically in microglia of aged mice (e.g., via intrathecal injection of AAV9-Cx3cr1-AHR) would provide critical evidence that restoring AHR function can ameliorate persistent hypersensitivity. These sophisticated genetic manipulations represent the logical next step to unequivocally establish causality and will be the focus of our subsequent investigations.

Finally, we should also acknowledge the limitations in terms of statistical power. Although our *in vivo* experimental data consistently demonstrated high statistical significance, the sample size of each group was relatively small at *n* = 8. Future studies that utilize larger cohorts and more conservative post hoc testing methods (such as Bonferroni correction or Tukey correction) will be able to further enhance the generalizability and robustness of these findings, particularly for secondary comparisons.

## 5 Conclusions

This study aims to investigate whether reduced expression of the aryl hydrocarbon receptor (AHR) in spinal dorsal horn microglia contributes critically to astrocyte overactivation and the prolonged persistence of hyperalgesia in the elderly. However, there are several limitations in this study. Firstly, the regulatory effect of microglial AHR on astrocyte inflammatory levels was verified solely at the cellular level, without interventions or verifications in animal models. Secondly, the precise mechanism by which microglial AHR expression regulates astrocyte inflammatory levels remains unclear. Previous research has indicated that activated microglia can regulate astrocyte activity by releasing transforming growth factor alpha (TGF-α), which binds to the epidermal growth factor receptor on astrocytes, and by releasing vascular endothelial growth factor b (VEGF-b), which interacts with the fms-related receptor tyrosine kinase 1 (FLT1) on astrocytes. It has been suggested that microglial AHR regulates microglial TGF-α and VEGF-b expression through direct effects on their promoters, as well as by limiting the activation of nuclear factor kappa B [16]. In the context of neuropathic pain, it remains unclear whether microglia regulate astrocyte inflammatory levels through TGF-α, VEGF-b, or other cytokines such as IL-1β, IL-6, and TNFα, and further experimental investigations are required to clarify these mechanisms.
